# A case for conducting business-to-business experiments with multi-arm multi-stage adaptive designs

**DOI:** 10.1057/s41270-022-00177-4

**Published:** 2022-08-10

**Authors:** Jonathan Legare, Ping Yao, Victor S. Y. Lo

**Affiliations:** Fidelity Investments, 245 Summer Street, Boston, MA 02210 USA

**Keywords:** Randomized controlled trial, Multi-arm multi-stage, Adaptive design, Simulation study, Experimental design, Business-to-business

## Abstract

Many businesses conduct experiments to scientifically test, measure, and optimize decisions in areas like sales, marketing, and operations efficiency. While randomized controlled trials (RCTs) or A/B tests are the dominant method for conducting business experiments especially for business-to-consumer marketing, adaptive designs have yet to make extensive inroads outside of the pharmaceutical and medical industries. In this study, we aim to raise awareness of the applicability and advantages of multi-arm multi-stage adaptive designs outside of clinical settings and we use simulations to demonstrate the value of these designs to modern business experiments, with a focus on business-to-business experiments such as testing alternative sales techniques. Our simulation results show that, compared to RCT, multi-arm multi-stage adaptive designs (MAMS) can reduce the sample size requirements and expected time to experiment completion whilst maintaining a similar level of statistical power. We also demonstrate that these benefits can translate into actual cost savings in conjunction with shorter time to market, resulting in higher overall efficiency over the traditional RCTs. MAMS serves as a strong alternative methodology in experiments where not all customers can be contacted at once such as business-to-business campaigns and general live channel programs which typically take weeks to months to complete.

## Introduction

Experimentation is an effective tool for testing new products or services and is utilized by several large companies (Fossett 2018; Wind 2018; Luca and Bazerman 2021). To mitigate risks when deploying innovative products or services, companies can conduct experiments, collect and analyze data, then make decisions based on the experimental results to ensure that new products or services provide value (Rissanen and Jürgen 2015). However, the costs, complexities, and technical knowledge requirements of running a traditional experiment can be extensive and may not have sufficient benefit for many businesses and applications.

### Traditional fixed designs

Randomized Controlled Trials (RCTs) are the classic experimental design, widely used for measuring the effectiveness of a novel treatment over a control, and prized for their robust, refined designs which help limit statistical biases (Bothwell et al. 2016). The core idea behind RCTs is to randomly allocate experimental units (e.g., patients) across treatment and control groups (e.g., drug and placebo). Allocating experimental units randomly to the treatment and control groups aims to avoid selection bias so that the resulting treatment effect (difference between treatment and control) can be attributed to the treatments rather than other variables that cannot be explicitly controlled (Kunz et al. 2007; Sedgewick 2015). In addition to testing and optimizing treatments for the overall population of interest, RCTs and related techniques can support development of uplift models to maximize treatment effectiveness at the subgroup level (Lo 2002; De Caigny et al. 2021; Haughton et al. In Press).

The conventional example of RCT design contains two arms: one treatment and one control; however, when researchers need to test multiple treatments, it may be more efficient to use a multi-arm RCT where two or more treatments share a single control arm. The advantages of multi-arm trials over classic two-arm trials are decreased sample size due to the shared control group; reduced administrative burden and lower costs from running a single experiment rather than conducting a separate two-arm experiment for each treatment; and, in the case of clinical trials, potentially benefiting patients through a higher chance of being assigned to a treatment group (Wason et al. 2014; Freidlin et al. 2008). As a typical illustration from the clinical setting, a four-arm randomized trail comparing laparoscopic and open hernia repairs (Hamza et al. 2010) concluded that laparoscopic techniques result in faster recovery and less postoperative pain, with only a single non-operative control rather than three two-arm experiments which would have required three control groups.

While the benefits of multi-arm designs are many, the caveat is that selecting suitable design parameters entails additional complexity such as the choice of multiple comparison corrections (Wason et al. 2014) to control false positives (Type 1 error), and selecting the optimal sample size allocation ratio among the various treatment arms and control arm (Wason & Thomas 2012). To facilitate design decisions and calculations for multi-arm experiments, leaders in this field developed a graphical web application and packages (Grayling & Wason 2020; Jaki et al. 2019) for the R programming language (R Core Team 2021).

While most published literature involving RCTs generally falls into clinical settings (Hartmann-Boyce et al. 2018; Dascal et al. 2017; Smith et al. 2010), randomized controlled trials have also been applied across many other domains, including marketing research by businesses and policy developers as illustrated by the following examples:

- A Canadian bookstore with an adjoining café employed a single treatment versus control randomized design to test whether administering an ambient chocolate scent within the bookstore resulted in increased sales of coffee and food items (McGrath et al. 2016).
- In the Indian state of Uttar Pradesh, researchers (Urpelainen & Yoon 2017) designed an RCT to test whether product demonstrations would increase awareness of solar technology and create markets for solar home systems. The results showed that marketing campaigns had no significant effect on sales or awareness of solar technology; rather, they discovered that access to credit from rural banks could be constraining the growth of solar home systems.
- To improve diet quality in minority neighborhoods, researchers from the Center of Obesity Research and Education at Temple University (Foster et al. 2014) designed a four-arm RCT to examine in-store product placement and promotion strategies and found that straightforward placement can significantly increase sales of healthier food and beverage items and thus could positively impact public health.

For further examples, see Field Experiments in Marketing (Simester 2017) with summaries of published field experiments in the marketing literature between 1995 and 2014 across areas including pricing, advertising and product development.

These examples of two-arm or multi-arm RCTs are called fixed designs where the number of sampling units (business days, villages, or supermarkets in the examples above) are essentially unchangeable once the experiment commences. Furthermore, the efficacy of each treatment arm is only estimated after the data for the entire sample is collected. As powerful and prevalent as they are, fixed designs suffer from various shortcomings such as predetermined fixed sample sizes, large sample size requirements, long study durations, and insufficient statistical power for measuring treatment effects in subgroups (Bhatt & Mehta 2016). These limitations along with the skyrocketing costs of drug development and clinical trials led to the adoption and development of adaptive designs (Chow & Corey 2011).

### Adaptive designs

Adaptive designs, in contrast to fixed designs, are more flexible and allow modifications to aspects of the study design after its initiation whilst maintaining the statistical validity and integrity of the experiment (Chow & Corey 2011). The core attractive component of adaptive designs is the ability to learn from accumulating data throughout an experiment and adjust important design parameters (e.g., effect sizes, sample size, number of treatment groups) where there may be uncertainty in the early design phase (Stallard et al. 2020). Experiments with adaptive designs commonly contain multiple stages (Chang 2014) based on time intervals or accrued sample sizes, and the criteria for design adaptations are predetermined based on the results of interim analyses that occur between stages of the experiment (Bhatt & Mehta 2016).

Many classes of adaptive designs appear in published and publicly available literature (Bothwell et al. 2018; van Werkhoven et al. 2019). Group sequential designs allow for early stopping of treatments arms (e.g., drugs) deemed effective or ineffective (or futile). In adaptive randomization designs the allocation of sample units (e.g., patients) to treatment arms may be adjusted during the experiment based on accumulated results. Sample size re-estimation designs allow the sample size to be adjusted based on improved estimates of treatment effect sizes. Adaptive dose-finding designs allow patients to be reallocated to doses with greater efficacy. Bayesian group sequential designs also use interim analyses but rather than estimating treatment effects they instead estimate the posterior probability that the null hypothesis is true based on the accumulated data (Berry 2006; Stallard et al. 2020). For detailed literature reviews, definitions, analysis, and implementations of various adaptive designs see (Chang 2014) and (Wassmer & Brannath 2016).

Several studies and research reviews show the benefits of adaptive designs over fixed designs (Bauer et al. 2016; Pallmann et al. 2018; Drazen et al. 2016). Given their advantages over fixed designs in many situations, adaptive designs have become popular within pharmaceutical companies for testing new drugs (Chang 2014). The clinical trials of COVID-19 vaccine candidates are a recent and critical application of adaptive designs with interim analyses and early stopping criteria (Polack et al. 2020; Mulligan et al. 2020).

Even prior to the completion of vaccine emergency use authorizations, a team of experts in the field of adaptive designs (Berry et al. 2020) published a cost benefit analysis of traditional and adaptive randomized clinical trials for COVID-19 vaccine candidates. Their study compared the expected duration of the trials and the expected number of infections and deaths, and they concluded that adaptive versions of vaccine efficacy RCT designs based on group sequential methods would provide greater net benefits than traditional (fixed) RCT designs, such as accelerating licensure with the FDA by several months, and fewer infections and deaths. Indeed, the clinical trial protocols published by the pharmaceutical companies Pfizer and Moderna both indicate use of case-driven adaptive designs with interim analyses (Polack et al. 2020; Mulligan et al. 2020).

The Moderna clinical study protocol (Moderna 2020; Baden et al. 2020) specifies two planned interim analyses to gather reliable evidence that the primary vaccine efficacy objective of 30% was achieved and to allow their data and safety monitoring board to recommend whether to pause or stop further enrollment due to efficacy or safety concerns. Interim analyses were planned to be performed after certain numbers of COVID-19 cases (infections) accrued, rather than at specific time intervals. Pfizer's protocol (Pfizer 2020) indicated the same vaccine efficacy objective of 30% but used four interim analyses based on case accruals and included early stopping criteria for futility if the predicted probability of achieving at least 30% vaccine efficacy at the final analysis was low.

Adaptive designs have been applied to clinical trials since the early 2000s, especially toward pharmaceutical research with the potential to accelerate drug development (Bauer et al. 2016). Only more recently have these designs been applied in other fields such as in the following examples:

- In human health outside of drug development, adaptive designs have been applied to behavioral modification intervention to increase physical activity. Using a two-stage design, participants who were unresponsive to a smartphone-based intervention in the first stage were rerandomized in the second stage and found that the addition of gamification increased their physical activity (Gonze et al. 2020).
- In the field of economics, researchers retrospectively applied two-stage group sequential adaptive designs to real data from framed and natural field experiments to illustrate how moving from single-stage RCTs to adaptive two-stage designs could improve the power of economic experiments (Jobjörnsson et al. 2021). Through a simulation study, they also showed that statistical power could be improved by two-stage designs without increasing the total sample size or cost of the study.
- A study aimed at combating more than half a billion pounds (GBP) of delinquent fines in the United Kingdom employed a multi-arm multi-stage adaptive design to test the effectiveness of text messages on inducing people to pay outstanding fines (Haynes et al. 2013). Text messages, which are inexpensive compared to dispatching bailiffs to collect fines, were found to significantly increase payments of delinquent fines, especially when addressing those who owe fines by name. Furthermore, the researchers concluded that [adaptive] randomized trials were effective at improving administrative efficiency.

### Multi-arm multi-stage designs

The class of adaptive design used in the delinquent fines study (Haynes et al. 2013) was Multi-Arm Multi-Stage (MAMS). MAMS is a class of adaptive designs which are based on group sequential designs that compare multiple treatment arms in a pairwise fashion with a single shared control arm over two or more stages and can make adaptive changes based on interim analyses (Wason et al. 2016; Ghosh et al. 2019). Like other adaptive designs, MAMS designs can employ early stopping of treatment arms due to efficacy or futility and other changes such as allocation of sample size to treatment arms, and they have been shown to improve experiment efficiency and reduce costs associated with testing multiple treatments in clinical trials (Sydes et al. 2009; Magirr et al. 2012; Wason et al. 2016; Ghosh et al. 2017). The MAMS package in R (Jaki et al. 2019) was developed to aid researchers with the design of MAMS studies across a variety of situations.

Simulation studies have been conducted to compare the performance of MAMS with other experimental designs in the traditional setting of clinical trials. A simulation study by Barthel et al. (2009) concluded that standard RCTs used for cancer trials would have realized significant savings in terms of accelerated trial time were they conducted using MAMS methodology, and that MAMS trials with three or more stages can yield greater savings due to increased probability of early stopping. Wason and Trippa (2013) made similar conclusions and added that adaptive methods are more ethical than fixed design multi-arm trials without interim analyses. Another study concluded that MAMS designs correctly identified the best treatment arm more often than fixed designs with the same total sample size (Bassi et al. 2021). A comparison of MAMS with 2 × 2 factorial designs (where patients are assigned treatments A, B, both or neither) found that MAMS designs are more robust and are expected to require smaller sample size and retain their statistical power compared with factorial designs (Jaki & Vasileiou 2017).

In clinical trial settings, multi-arm, multi-stage designs are valuable because experiments tend to have long durations, high costs, and high potential risks. But when would a business or marketing research team benefit from using MAMS? For most consumer research and business-to-consumer (B2C) experiments where the subject population is large, where experimental unit costs and potential risks are low, where data can be gathered rapidly in a day or so, and where online crowdsourcing services like Qualtrics and Amazon MTurk have made consumer subjects increasingly accessible at low cost (Arndt et al. 2022; Goodman & Paolacci 2017), MAMS designs likely will not provide sufficient value over RCT designs to justify their use. For business-to-business (B2B) research applications, however, we expect MAMS to realize cost and time benefits over RCT designs due to common challenges inherent with B2B experiments such as smaller sampling populations, more complicated random allocation of experimental units (aka companies) to treatment and control groups, an absence of cost-effective data sources, and higher complexities of decision-making processes and IT systems (Simester 2017; Thompke 2020; Viglia et al. 2021; Kienzler & Kowalkowski 2017).

The aim of this study is to demonstrate the applicability of MAMS in a business-to-business (B2B) setting and to increase the awareness of adaptive designs outside of clinical trials. In this paper, we use simulations to show the advantages of MAMS over fixed RCTs. We show that the statistical strength of both approaches is similar. In the worst-case scenario where no treatments stop early, RCT has a small advantage over MAMS in terms of sample size. In all other cases MAMS has a significant advantage. We use these results to make inferences about the potential costs, tradeoffs, and advantages in a business setting. Finally, we outline the hypothetical cases where MAMS can save a business time and money over RCTs based on our results.

## Methods

To estimate the expected sample sizes and expected powers of RCT and MAMS designs, we performed simulations using the *MAMS* package (Jaki et al. 2019) for the R programming language (R Core Team 2021). This section describes how we used these tools to generate our results, and we have also included a generalization of our methodology in the appendix.

### Simulation settings

Across all simulations and all scenarios, we fix several experimental design and simulation parameters. The familywise error rate (FWER), which is the probability of mistakenly rejecting at least one true null hypothesis,^1^ is controlled such that FWER ≤ α, with α = 0.05. The desired power, which is the probability of correctly rejecting the null hypothesis when the alternative hypothesis under the least favorable configuration is true, is controlled such that POWER ≥ 1 – β, with 1 – β = 0.90. For both single- and multi-stage designs, we assume a single, shared control arm which we compare with *K* = 4 treatment arms. For multi-stage designs we use *J* = 3 stages. Experimental units are assumed to be independent and identically distributed Bernoulli random variables. In both designs we randomly and equally allocate sampling units between treatment arms and across stages. Each expected sample size and expected power calculation is based on 100,000 simulations. For reproducibility of results, we set the random number generator seed to 12,345 prior to executing each batch of simulations.

### Experiment design and scenarios

In this study, we estimate the expected sample size and power for a variety of scenarios defined by two parameters: the baseline success probability *p*_0_ and an interesting treatment effect size δ. For example, *p*_*0*_ could represent a business-to-business sales team's success rate at converting prospects into customers using a conventional sales technique, i.e., conversion rate. For industries with longer sales cycles that span multiple quarters or even years, such as enterprise software, the “success” measure could be based on an intermediate milestone rather than a closed sale, such as a customer scheduling a sales appointment or expressing interest in a purchase. In the context of cross-selling or upselling to existing customers, *p*_*0*_ can represent the baseline success rate for selling an additional product or a higher-end product to business customers.

There are many alternative techniques for sales and marketing, including but not limited to the following dimensions:

1. Who—What type of sales representatives would be the best for each product and for each type of customer? How many salespeople are needed?
2. What—What product features are we focused on? What is the appropriate communication script? How much pricing discount can we offer? What incentives can we introduce during the sales cycle?
3. When—What is the right timing (e.g., day of the week) and frequency of interactions?
4. How—What combination and sequence of channels should be considered (e.g., email followed by phone call followed by visit, or LinkedIn interaction followed by video call)?

The above dimensions can lead to many alternative sales and marketing techniques that are empirically testable in randomized experiments (Hanssens 2015). In our simulation, suppose the team has *K* alternative sales techniques (e.g., driven by different channels and pricing) to try with unknown success rates *p*_*1*_, … *p*_*K*_ and the team wants to detect with high probability any technique where *p*_*i*_ – *p*_0_ ≥ δ, where δ is the threshold for incremental success rate that is considered business-meaningful, i.e., “interesting” treatment effect. We simulated four values of *p*_*0*_ (0.10, 0.30, 0.50, and 0.70) and eight values of δ (0.02, 0.05, 0.10, 0.15, 0.20, 0.25, 0.30, and 0.40).

For each design and scenario, we estimate the expected sample size under two conditions:

(1) under the global null hypothesis where no treatments have any effect:

H_0*1*_: *p*_*0*_ = *p*_*1*_, … H_0K_: *p*_*0*_ = *p*_*K*_*.*

And (2) under the *least favorable configuration* (LFC) wherein exactly one treatment arm has a non-zero interesting treatment effect of size δ*,* and all other arms are ineffective with effect size 0.

H_0*1*_: *p*_*1*_−*p*_*0*_ = δ, … H_0K_: *p*_*0*_ = *p*_*K*_*.*

We also estimate the expected power under the least favorable configuration for each design and scenario.

### Required sample sizes and critical bounds

For each scenario, the first step in the simulation process is to compute the *required sample size* for both designs along with the lower and upper bounds for determining futility and efficacy of treatments at each interim analysis phase of the multi-stage design. To compute them, we use the *ordinal.mams* function (Jaki et al. 2019) with the following input parameters: The global parameters such as the one-sided familywise error rate, power, sample size allocation ratios, number of treatment arms, and number of stages are defined as discussed earlier. For the vector of expected probabilities of success and failure under control conditions we use (*p*_0_, 1 – *p*_0_). The interesting treatment effect odds ratio is computed as [*p*_*1*_ (1 – *p*_0_)] / [*p*_0_ (1 – *p*_*1*_)] where *p*_1_ = *p*_0_ + δ, and the uninteresting treatment effect odds ratio is set equal to one to signify equal odds. The upper boundary shape follows the design of O'Brien and Fleming (1979) while the lower bound is constant and set to zero. The number of quadrature points used to determine the fineness of the numerical integral approximation is set to its default value of 20.

### Simulations to estimate expected sample sizes

The second step in the process is to simulate RCT and MAMS designs to estimate expected sample sizes, and we accomplish this using the *mams.sim* function (Jaki et al. 2019). Most function arguments are based on outputs of the *ordinal.mams* function, including the vectors of upper and lower boundaries and the sample size matrix which is computed as the product of the allocation ratios and the required sample size.

The *ordinal.mams* function requires us to specify the vector of interesting treatment effects which contains *K* elements, one per treatment arm, and where each element corresponds to the probability that a randomly selected observation from treatment *k* has a better outcome than a randomly selected observation from the control group. In our simulations, we set *K* = 4 treatment arms plus the control group. Under the global null hypothesis, the treatment and control groups are assumed to be equally effective, meaning that our vector is simply (0.5, 0.5, 0.5, 0.5). Under the least favorable configuration, only the first treatment is assumed to have a non-zero treatment effect, meaning that we use the vector ($\nu$, 0.5, 0.5, 0.5) where $\nu$ is computed as

1 $$\nu =\Phi \left(\frac{\delta }{\sqrt{2{\sigma }^{2}}}\right)=\Phi \left(\frac{\delta }{\sqrt{2{p}_{0}\left(1-{p}_{0}\right)}}\right)$$

with $\Phi$ representing the cumulative density of the standard normal distribution. This equation is from (Jaki et al. 2019) and we substitute ${\sigma }^{2}={p}_{0}\left(1-{p}_{0}\right)$ since our sampling units are assumed to be Bernoulli distributed with mean ${p}_{0}$ and variance ${p}_{0}\left(1-{p}_{0}\right)$.

Note that our simulation design calls for the use of binary endpoints since we assume our experimental units are drawn from a Bernoulli distribution, and accordingly we use the *ordinal.mams* function which supports binary and ordinal endpoints. While this may address most business applications in our experience, some business studies may require nonbinary outcomes and indeed MAMS designs allow for binary, ordinal, Gaussian, and time-to-event data to be used as endpoints (Wason 2019), and the MAMS Package in R (Jaki et al. 2019) contains functions to support these nonbinary designs.

### Simulations to estimate power

The third step in the process is to simulate RCT and MAMS designs to estimate the power for a fixed sample size, *n*. For this we again use the *mams.sim* function (Jaki et al. 2019) with the same parameters and settings as used for the expected sample size calculations except for the sample size allocation matrix. For the RCT simulations we sample equally over the five arms (four treatments and a control), and for the MAMS simulations the allocation matrix is cumulative and splits the sample equally over the five arms and three stages. Mathematically, the allocation matrices are computed as

2 $${N}^{RCT}=\left[ \begin{array}{cc}\frac{n}{\left(K+1\right)}& \cdots \end{array} \right] \hspace{2em} {N}^{MAMS}=\left[\begin{array}{c}\begin{array}{cc} \frac{1n}{J\left(K+1\right)}& \cdots \\ \frac{2n}{J\left(K+1\right)}& \cdots \\ \frac{3n}{J\left(K+1\right)}& \cdots \end{array}\end{array}\right]$$

where *N*^*RCT*^ and *N*^*MAMS*^ have dimensions (1 × 5) and (3 × 5), respectively, each with repeating column vectors, and where *J* and *K* represent the number of stages and treatment arms, respectively, and are set to *J* = 3 and *K* = 4 in our simulation. Note that the allocation matrix for MAMS is cumulative, meaning that each row vector of *N*^*MAMS*^ equals *N*^*RCT*^ multiplied by the row index (stage number) divided by the number of stages. The power calculations are repeated for a range of sample sizes: 150, 300, 600, 900, 1200, 1800, 2400, and 3000.

### Simulations to estimate impact of treatment effect assumption errors

In this section we assume that the true treatment effect $\updelta$ is unknown during the design phase of the experiment and instead we use an estimated treatment effect ${\updelta }^{\mathrm{^{\prime}}}=\updelta +\upvarepsilon$ where $\varepsilon$ represents the treatment effect assumption error. The objective is to measure using simulations the impact of this treatment effect assumption error on the expected sample size and power. To accomplish this, we follow the simulation methods hereinabove except that we use sample size allocation matrices based on the assumed treatment effect size ${\updelta }^{\mathrm{^{\prime}}}$ and we compute the vector of interesting treatment effects under the least favorable configuration using the true treatment effect size $\updelta$.

More specifically, to estimate the required sample sizes for RCT and MAMS designs, we follow the procedure for computing required sample sizes (see Required sample sizes and critical bounds) using *ordinal.mams* except that in place of $\updelta$ we substitute ${\updelta }^{\mathrm{^{\prime}}}=\updelta +\upvarepsilon$ when determining the parameters to that function. These required sample sizes based on ${\updelta }^{\mathrm{^{\prime}}}$ are then substituted into the numerators of the sample size allocation matrices described in Eq. 2 of the previous subsection. For the vector of interesting treatment effects, we perform the same calculation described in Eq. 1 above using the true treatment effect size $\updelta$. We then simulate the designs under the least favorable configuration using *mams.sim* and record the expected sample size and power. We repeat this procedure for various levels of $\varepsilon$, and then compute the difference in expected sample size and power using $\upvarepsilon =0$ as the baseline for comparison. The resulting differences represent the change to sample size and power corresponding to the treatment effect assumption error $\upvarepsilon$.

## Results

### Required sample size

**Fig. 1:**
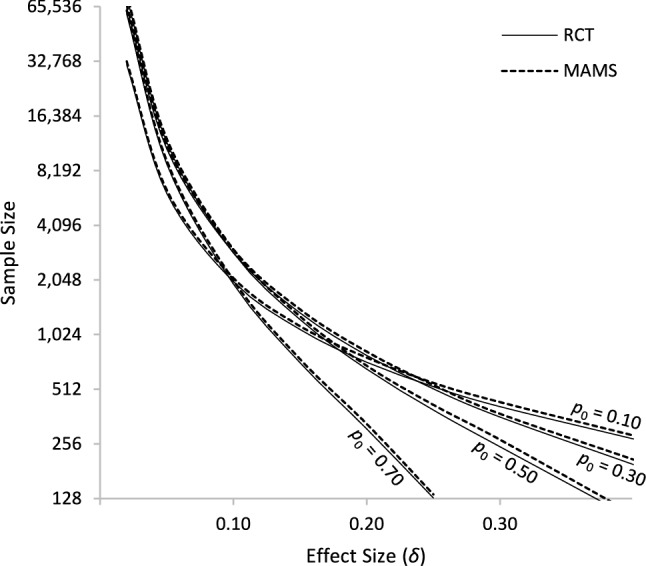
Required Sample Size by Treatment Effect Size. *Note.* The required sample size for MAMS designs is consistently 4% to 9% higher than for RCT designs across multiple success probabilities *p*_0_ and estimated treatment effect sizes δ

The *required sample size* is the initial estimate of the number of sampling units needed for an experiment. It is primarily determined by study design parameters α and β to control the probabilities of committing Type I and Type II errors, respectively, and the assumed treatment effect size δ. Holding all other factors constant, an experiment that aims to detect a smaller treatment effect will require a larger sample size. As a proxy for required sample size, we used the *expected sample size* across 100,000 simulated RCT and MAMS experiments (as described in *Methods*) for varying levels of success probability (*p*_0_) and estimated treatment effect sizes (δ). The results are summarized in Fig. 1.

As expected, for given levels of *p*_0_, we observe an inverse relationship between estimated treatment effect size and required sample size. Moreover, we observe that MAMS designs consistently require higher sample sizes than RCT designs where no treatments stop early (the *expected* sample sizes can be lower for MAMS, as we explain later); however, the required sample size differences are proportionally small. For example, with *p*_0_ = 0.10 and a small effect size δ = 0.05, the required sample sizes of MAMS and RCT designs are 6,420 and 6,146 with a ratio of 1.04. Similarly, with *p*_0_ = 0.10 and a larger effect size δ = 0.40, the required sample sizes are 285 and 273, respectively with the same 1.04 ratio. Across all success probabilities (*p*_0_) and treatment effect sizes (δ) we observed the required sample size of MAMS designs in the worst-case scenario where no treatments can be stopped early to be 4% to 9% greater than RCT designs.

### Expected sample size

While the *required sample size* is determined during the design stage, the *actual sample size* needed by MAMS designs in practice may be lower, since each of the interim analyses between experiment stages may lead to early stopping of treatment arms due to efficacy or futility.

**Fig. 2:**
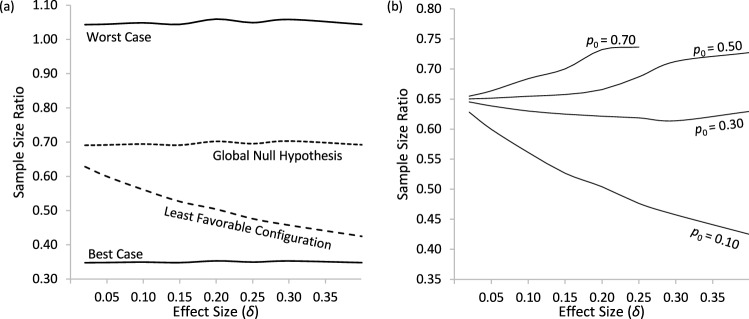
Ratio of MAMS to RCT Expected Sample Size for **a** baseline success probability p_0_ = 0.10, and **b** for various baseline success probabilities under the least favorable configuration. *Note.* The middle two curves in **a** represent the ratio of the expected sample sizes under the global null hypothesis and least favorable configuration, whereas the best- and worst-case lines represent the minimum and maximum ratios of expected sample sizes observed across all 100,000 simulations. The topmost ratio curve in **b** for *p*_0_ = 0.70 is shorter because the effect size δ must be less than 0.30

The worst case for sample size in a multi-stage experiment is that no treatment arms are deemed effective or futile during interim analysis phases and the best case is that all treatment arms are stopped early before the second stage of the experiment. In our simulations, we observed MAMS designs require 1.04 to 1.09 times the sample size of RCT designs in the worst case, and only 0.35 to 0.36 times in the best case. These cases correspond to the top and bottom lines of Fig. 2(a).

With respect to expected sample size, our simulation results show that MAMS designs are consistently more efficient than RCT designs. Under the global null hypothesis, where no treatments are effective, we observe that MAMS designs on average used about 70% of the sample size of RCT designs. And under the least favorable configuration where only one treatment is effective, we observe that MAMS used between 42% to 74% of the sample size on average, and that the sample size savings is dependent upon the treatment effect size δ. These two cases are illustrated in the middle two lines of Fig. 2(a). With a small baseline success probability *p*_0_ = 0.10, the sample size savings tend to increase with increasing effect size δ. This relationship does not hold for larger success probabilities, as shown in Fig. 2(b) and we observe diminishing sample size savings for larger baseline success probabilities.

### Power

**Fig. 3:**
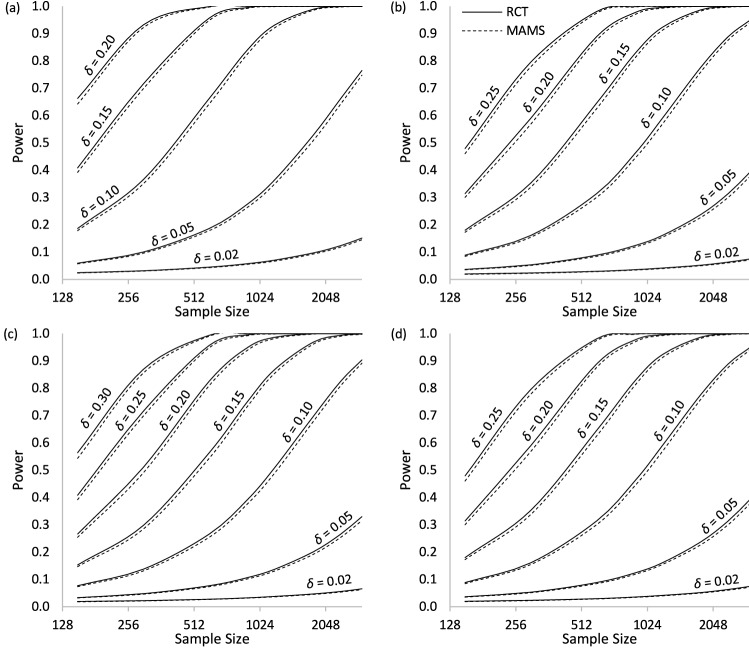
Expected Power, by Sample Size. *Note.* Shown for baseline success probabilities: **a** *p*_0_ = 0.10, **b** *p*_0_ = 0.30, **c** *p*_0_ = 0.50, and **d** *p*_0_ = 0.70

For each scenario (*p*_0_, δ), we estimated the power of each design under the least favorable configuration across a variety of sample sizes ranging from 150 to 3000 while holding all other design variables constant. The results of our simulations are plotted in Fig. 3, and the plots show remarkably similar power curves between the two designs, with RCT designs consistently having negligibly higher power than MAMS designs. The median difference is only 0.003 in favor of RCT designs. The power curves are steeper for larger effect sizes, since treatments with larger effect sizes are easier to detect.

### Misestimation of treatment effects

**Fig. 4:**
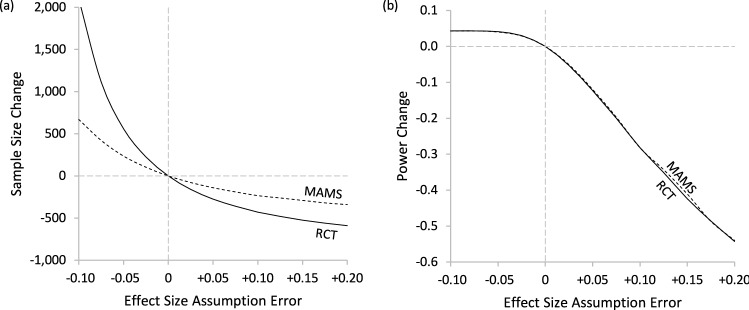
Expected Sample Size change and power loss, by Effect Size Assumption Error under LFC. *Note*. The x-axis indicates the amount the treatment effect is under- or over-estimated and the y-axis measures the corresponding amount of additional: **a** sample size required or saved and **b** power gained or lost due to the misestimation of the treatment effect. These plots assume success probability *p*_0_ = 0.30 and true effect size δ = 0.20 although the directionalities and relationships hold for other parameter values, meaning that underestimating δ increases the expected sample size more for RCT designs than MAMS designs and while overestimating δ decreases the sample size, both designs suffer sharply and approximately equally from reduced power

For RCT and MAMS designs, a key determinant of sample size is the initial estimate of the treatment effect size δ*.* Our simulation results thus far implicitly assume the experimenter accurately guessed the treatment effect size δ. However, in practice this is unrealistic since if one knew the treatment effect confidently and accurately beforehand then there would be little need for an experiment. To understand the tradeoffs associated with treatment misestimation, we simulated expected sample size and power across a range of treatment assumption errors. The results are presented in Fig. 4 which shows the incremental gain/loss to required sample size and power by the assumption error in the treatment effect.

For both designs, underestimating the effect size during the design phase will increase the required sample size and the power of the experiment; however, the expected increase in sample size due to this effect size underestimation will be substantially less for MAMS designs than for RCT designs. As shown in Fig. 4(a), when the baseline success rate is *p*_0_ = 0.30 and the true effect size is δ = 0.20 and we assume the least favorable configuration, then if we underestimate δ by 0.05, the RCT design will require an additional 560 sampling units. In contrast, MAMS will require 585 additional sampling units at the design phase, but the expected sample size will only increase by 235 units, so the expected increase in sample size of underestimating the treatment effect is considerably less with MAMS designs than RCT designs. Similarly, the decrease in sample size of overestimating the treatment effect δ is a substantial loss of power, with RCT and MAMS designs losing nearly equal amounts as illustrated in Fig. 4(b).

### Cost analysis

To measure the experimental cost of MAMS versus RCT, we assume a $1,000 cost per sampling unit. The cost simulation is set up as a B2B marketing campaign case (Wang et al. 2019), and the cost-savings are derived from the flexibility of adaptive design, which allows interim analysis to drop the ineffective treatment arms or to stop for efficacy of interventions. Table 1 summarizes the results of the experimental cost comparison between RCT and MAMS designs for two scenarios, each under the worst case, best case, and least favorable configuration (LFC). The rightmost column of Table 1 shows the expected cost saving (or loss) of MAMS compared to RCT designs. In the scenario where *p*_0_ = 0.3 and $$\updelta$$= 0.3, the simulation results indicate that MAMS would save $139 K (39%) compared to RCT under the least favorable configuration and $235 K (65%) under the best case. MAMS would cost $15 K (4%) more than RCT under the worst case. In all other scenarios that we tested, we found that MAMS results in cost savings over RCT designs under LFC and best-case conditions.

**Table 1:** Experimental Cost Comparison between MAMS and RCT

	RCT		MAMS			
Scenario	Sample Size	Cost	Case	Expected Sample Size	Expected Cost	Expected Cost Savings (%)
*p*_0_ = 0.3, δ = 0.3	360	$360 K	Worst	375	$375 K	− $15 K (− 4%)
			LFC	221	$221 K	$139 K (39%)
			Best	125	$125 K	$235 K (65%)
*p*_0_ = 0.3, δ = 0.1	2892	$2,892 K	Worst	3,015	$3,015 K	− $123 K (− 4%)
			LFC	1,822	$1,822 K	$1,070 K (37%)
			Best	1,005	$1,005 K	$1,887 K (65%)

Adding a moderate per-stage cost to the simulations (e.g., $10k–50k per stage) would lower the magnitude of the cost savings but retain the overall directionality of the results

## Discussion and conclusion

Businesses have traditionally used RCTs or A/B tests to conduct experiments to scientifically inform decisions in areas like marketing strategies, product development, operations analysis, etc. (Fossett 2018; Wind 2018; Luca & Bazerman 2021). In this paper, we present a comparison of RCT and MAMS and show that MAMS can reduce the expected number of sampling units and expected time to experiment completion while maintaining a similar level of statistical power.

Under what scenarios could MAMS designs have an advantage over RCT? Since MAMS is a form of sequential experimentation where we can draw conclusions at earlier stages via interim analyses, the key enabler for cost savings is that subjects are not all contacted initially. Thus, if an interim analysis determines that the experiment can stop earlier, there is no added cost associated with additional (unneeded) experimental units. Most business-to-business campaigns (which often involve a combination of contact channels and multiple human interactions) with a long response time and some live channel business-to-consumer programs which take a longer cycle to complete (say, weeks, months, or longer) satisfy such criterion. On the other hand, in business-to-consumer experiments such as those conducted online or by email where millions of customers can be contacted almost at once with their responses gathered digitally in short period of time, there is naturally limited or no opportunities for MAMS. However, when this criterion is met, the efficiency of MAMS designs can lead to business advantages such as cost savings or improved time to market, but the expected savings will depend upon several cost drivers. In a general sense, the total cost of an experiment will depend on fixed initial design and setup costs, per-unit costs associated with each sampling unit, and per-stage costs associated with executing each stage of the experiment (e.g., sample selection, data analysis).

When per-stage costs are low relative to per-unit costs, then the decreased expected sample size of MAMS is likely to be more cost-effective than RCT for the same statistical power. A sales department in a small business that wishes to test different outbound call scripts could benefit from a multi-stage design if it would require substantial time of a salesperson to prepare, call the prospective customer, and then manually record results for later analysis. By stopping ineffective treatments early, the sales department could eliminate unnecessary additional outbound calls and instead focus on higher value activities; furthermore, they would decrease the opportunity costs associated with using a demonstrably ineffective sales technique with prospective customers.

In some cases, a multi-stage design may be more costly than a single-stage design, especially when per-stage costs are high (for example, due to high experimental setup costs) and per-unit costs are low. Consider an experiment in a large company where multiple business departments (e.g., marketing, sales, analytics, technology, senior management) must coordinate prior to executing each stage of an experiment to measure the effectiveness of various outbound email campaigns. The business coordination and planning costs of each stage would likely dwarf the costs of sending any number of emails to customers. A single-stage (RCT) design in this case may be more cost-effective unless the researchers are relatively certain that one or more treatment effects will be large enough such that a multi-stage experiment will benefit from early stopping.

Other factors to consider with MAMS designs are the number of treatments and number of stages which are both driven by business requirement and constraints. In general, testing more treatments (e.g., sales techniques or pricing strategies) enables us to learn more and arrive at a more “optimal” solution, similar to the regular RCT settings in this case. The number of stages depends on how long the experiments can run. The reason why COVID-19 vaccines benefited tremendously from adaptive design is because it took a prolonged period (several months) to recruit patients and complete the experiments, so having multiple interim analyses allow pharmaceutical companies to draw conclusion earlier with a clear benefit to public health.

Additionally, the cost of the experiment is not the only cost – time to market and lost business opportunities present a cost as well. Again, if one or more treatment effects are large enough that early stopping is likely and the time to measure each experimental unit is costly, then MAMS may have a business advantage even if the pure financial cost of the experiment is expected to be higher than with RCT. For a start-up where time to market is a vital factor, for example, this advantage could outweigh the higher per-stage financial costs. For example, assume a business is running a 6-month sales campaign experiment. With a RCT design, a business decision is only made at the end of sixth months. However, with a 3-stage MAMS design, there are two interim evaluations: the first one at the end of the 2^nd^ month, and the second one after the 4th month. If there are significant treatment effects identified at first and second interim evaluations, a business decision can be made earlier and there would be corresponding cost savings and potentially additional revenue benefits by going to market faster with the winning sales strategy.

In a business setting, it is less common to have an accurate estimate of the treatment size prior to conducting an experiment. Misestimation of treatment effects has implications for the experiment: underestimation increases the required sample size and overestimation decreases the statistical power. However, because of the lesser penalty (in terms of sample size) with adaptive designs, the experimenter can instead focus on the threshold for business value, i.e., the minimum meaningful value from the business perspective, instead of attempting to accurately estimate treatment effect sizes. If the threshold for business value is lower than the true treatment effect size, then MAMS will likely stop early, resulting in cost savings (unlike an RCT). In the case where treatments are ineffective (i.e., global null hypothesis), the sample size penalty is typically less with MAMS.

**Fig. 5:**
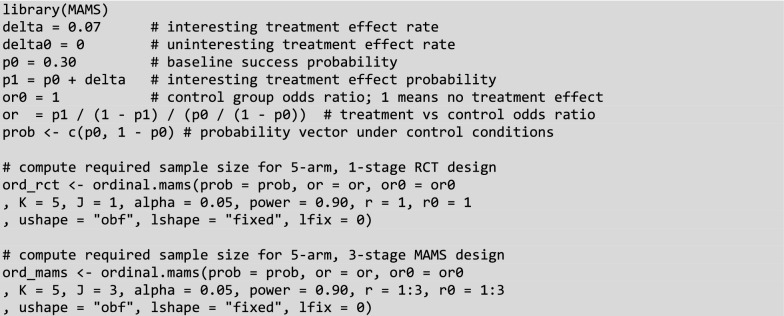
Sample R Code to Compute Sample Size for RCT and MAMS Designs. *Note.* Relatively little code is required to compute the required sample size for RCT or MAMS designs using the *MAMS* package (Jaki et al. 2019)

Given the advantages of adaptive designs, the inevitable question is why they have not been more adopted by businesses. Awareness could be a reason since, to the best of our knowledge, most of the research and implementation has been published in scientific and medical literature. Given the specific nature of the medical applications, the business advantages of adaptive designs may not be apparent. Another possible explanation is the perceived difficulty of implementation. While the theoretical basis of MAMS is certainly more complex than RCT, in practice both methods can be similarly implemented using the MAMS R package (Jaki et al. 2019), as we show in Fig. 5. The code for determining the required sample size is nearly identical for RCT and MAMS, with the only difference being the number of stages specified. Alternatively, there are web applications that will calculate the required sample size for RCT and adaptive designs, such as (Grayling & Wason 2020). Any researcher who is comfortable with RCT need not be intimidated by MAMS.

The adaptive design MAMS has comparable statistical power to RCT across many scenarios and in many cases can save money or provide other business advantages compared to RCT. Despite its theoretical complexity, MAMS implementation in practice is similar to RCT and simulations can be performed if desired to determine whether RCT or MAMS is more appropriate prior to a large investment in an experiment. Adaptive designs have made significant inroads in clinical trials due to their many advantages (Chow & Corey 2011) and by writing this article, we aim to make MAMS more approachable in a business setting.

## Appendix: A guide to conduct MAMS experiments

### Design phase

1. Define the business goal and available treatments and specify the number of treatment arms.
2. Estimate treatment effect sizes based on data or knowledge or decide on treatment effect size thresholds required for meaningful business value.
3. Estimate the required sample size for a single-stage design. This requires the researcher to specify the Type I and Types II error rates, and to decide whether to use equal allocation of sampling units across treatment groups.
4. Determine the appropriate number of stages. This may be based upon the estimated sample size and other business constraints and parameters such as the expected duration of the entire campaign and each stage.
5. Determine the final required sample size for a MAMS design along with the lower and upper bounds for determining futility and efficacy of treatments at each interim analysis phase.

### Experiment phase

1. Recruit the minimum number of sampling units for the current stage of the experiment and then randomly allocate them across the treatment and control arms.
2. Execute the experiment and gather data.
3. Analyze the cumulative dataset and determine whether treatment arms can be dropped due to efficacy or futility.
4. Repeat steps (1) to (3) for each stage of the experiment, or until all treatment arms have been dropped due to efficacy or futility, whichever is achieved first.

## Footnotes

1 Generalized from the typical Type I error for a single hypothesis.
